# Preliminary accuracy, inter- and intra-rater reliability of a 4-point cervical artery dissection diagnostic decision support tool in an ambulatory setting using real and simulated cases: a quality improvement project

**DOI:** 10.1186/s12998-026-00679-3

**Published:** 2026-10-11

**Authors:** Tyler H. Snodgrass, Zachary A. Cupler, Christopher J. Farrell, Lindsay R. Rae

**Affiliations:** 1 VA Finger Lakes Healthcare System, Canandaigua, NY USA; 2 Butler VA Health Care System, Butler, PA USA; 3 Geriatric Research Education and Clinical Center (GRECC), VA Pittsburgh Healthcare System, Pittsburgh, PA USA

**Keywords:** Cervical artery dissection, Diagnostic decision support tool, Diagnostic accuracy, Inter-rater reliability, Intra-rater reliability, Quality improvement, Simulation

## Abstract

**Background:**

Cervical artery dissection (CeAD) is a rare but life-threatening condition that commonly presents with non-specific symptoms such as neck and head pain, complicating early recognition in ambulatory care settings. The CeAD diagnostic decision support tool (DDST) is a 4-item assessment designed to identify patients that should be referred for advanced diagnostic work-up to rule out CeAD; however, its utility in ambulatory settings has not been established.

**Methods:**

This quality improvement project aimed to evaluate the accuracy, inter-rater reliability, and intra-rater reliability of chiropractors applying the CeAD DDST using clinical vignettes simulating a typical ambulatory encounter before deploying the DDST broadly as part of the clinical workflow. Five experienced chiropractors assessed 40 standardized clinical vignettes. CeAD-positive cases (*n* = 10) were derived from published imaging-confirmed ambulatory care case reports. CeAD-negative cases (*n* = 30) were developed using generative artificial intelligence and refined by senior authors to reflect common conditions encountered in chiropractic practice. Raters independently assessed cases with the DDST (score 0–4), with a cutoff score of ≥ 3 recommending referral for advanced diagnostic work-up in two rounds, beginning November 11, 2025 and January 07, 2026, with a two-week capture period. Accuracy, inter-rater reliability, and intra-rater reliability were assessed.

**Results:**

Accuracy in identification ranged from 72.5% to 95.0% across raters and rounds. False positives and negatives were present across raters in both rounds, with false positives being more prevalent. Inter-rater reliability was moderate to substantial in both rounds (Round 1: κ = 0.609, 95% CI 0.432–0.806, Round 2: κ = 0.540, 95% CI 0.365–0.735). Intra-rater reliability ranged from moderate to almost perfect agreement (range 0.457–0.931).

**Conclusions:**

The CeAD DDST demonstrated moderate to good accuracy and reliability among chiropractors in a simulated ambulatory setting. The presence of false negatives and inter-rater reliability falling below recommended benchmarks preclude recommendation for implementation at this time. Iterative refinement informed by end-user input and prospective ambulatory testing is warranted prior to clinical adoption of the CeAD DDST. Future evaluations should include prospective studies using real clinical data to better represent real-world settings.

**Supplementary Information:**

The online version contains supplementary material available at https://doi.org/10.1186/s12998-026-00679-3.

## Background

Cervical artery dissection (CeAD), inclusive of the vertebral artery and carotid artery, is a rare, life-threatening condition. Defined as a tear in the intima or rupture of the vasa vasorum, CeAD can occur in the presence or absence of trauma, is the most common cause of stroke among younger adults, and is a complex interplay between comorbidities, environmental exposures, genetic or congenital predispositions, and anatomic considerations [1–3]. Spontaneous CeAD incidence was reported at 4.69 per 100,000 person-years from 2002 to 2020, with a near four-fold increase in incidence rate over that time period [4]. The most common early presentation of a CeAD is head and/or neck pain. [1] Further clinical manifestation differentiates between vertebral or internal carotid artery involvement with neck pain being more common among individuals experiencing a vertebral artery dissection compared with internal carotid artery dissection [5].

CeAD may progress to ischemic stroke or transient ischemic attack, resulting in neurological symptomology localized to the impacted cerebral area classified, such as anterior or posterior circulation stroke syndromes or lateral medullary infarction [6]. CeAD alone may present without neurological symptoms, though cranial neuropathy, cervical radiculopathy, and Horner syndrome have been shown to manifest in a subset of CeAD cases [6–8]. Early CeAD may be underrecognized given the non-specific, subtle prodromal symptom features. Because of the sudden, evolving nature of the condition, early recognition of CeAD is vital for appropriate diagnosis and escalation of care for improved outcomes [9]. Reliable, early identification of CeAD in ambulatory, outpatient settings remains a diagnostic dilemma due to the evolving nature of cerebrovascular events, overlapping, non-specific prodromal symptoms, and lack of immediate access to advanced diagnostics. This ongoing diagnostic dilemma during the early stages of CeAD progression justifies the utility in development of CeAD screening tools or point of care imaging modalities that improve early detection and management of CeAD through objective criteria and risk stratification [2].

Neck pain and headache are common symptomatic presentations that are not unique to CeAD. The age-standardized point prevalence of neck pain is projected to increase by 32.5% over the period from 2020 to 2050 from 203,000 to 269,000,000 people globally. [10] These common, non-specific symptoms often prompt a patient to seek care initially from ambulatory care providers [11, 12]. Without concerning neurological symptoms associated with evolving CeAD, such as dizziness, visual disturbances, or other obvious neurological changes, CeAD remains difficult to discern from other more common etiologies of neck pain and headache (e.g., primary musculoskeletal) [13, 14].

The first line imaging techniques recommended for CeAD are computed tomography angiography (CTA) or magnetic resonance angiography (MRA), with the gold standard being digital subtraction angiography (DSA) [15, 16]. Both CTA and MRA have varying sensitivity and specificity for diagnosing CeAD when compared to DSA, with CTA ranging between 65% and 100% and MRA ranging between 30%-100% [15]. Accessibility to CTA, MRA, or DSA is a limited resource in most healthcare settings outside of tertiary hospital centers. Given ambulatory care providers are often practicing outside of a tertiary care setting, immediate use of advanced imaging services, beyond plain film radiography, is comparatively low [11, 12, 17]. In addition to limited access, routine screening for CeAD for all cases of non-specific neck and/or head pain with advanced diagnostic imaging is a costly and an inefficient use of system resources. Routine diagnostic testing increases healthcare cost, utilization, and burden to patients (e.g., time, psychological stress, delay in care), especially for primary musculoskeletal conditions where imaging in acute, non-specific neck pain is discouraged in the absence of red flags [18, 19].

A variety of barriers may inhibit clinical decision-making to escalate a patient for further diagnostic work-up, including procedure standardization within and between health care systems, provider education and training, regional resources, provider experience and confidence, patient self-reporting, condition presentation variability, and availability of staff or resources [20, 21]. Diagnostic decision support tools (DDSTs) may aid in the gathering of standardized objective findings and risk stratification for prioritization of ‘high-risk’ patients for which care should be escalated. This may be especially useful in ambulatory care settings where resources may be limited and cost and burden may outweigh routine screening with advanced testing. DDSTs improve guideline-concordant care and optimize patient safety (e.g., prescriber adherence to guidelines in ambulatory care settings) [22]. Recent efforts to reduce adverse events in the evaluation and management of cervical spine conditions reprioritized the necessity to recognize the potential for evolving pathology (e.g., CeAD) [23–25]. Reducing barriers through utilization of a DDST sensitive for patients at increased risk for CeAD may improve earlier and more accurate diagnosis, promote care standardization, enhance clinician diagnostic confidence, and streamline appropriate care escalation.

Thomas et al. [14, 16] devised, refined, and validated a 4-item CeAD DDST for use with patients who present with neck pain and/or headache based on common clinical features of patients with confirmed CeAD. Validated with a prospective case-control design through emergency department admissions, the CeAD DDST attempts to support clinicians in the detection and triage of those patients at moderate or high risk of CeAD most appropriate for further advanced diagnostics work-up [14, 16]. To date, this DDST has not been widely adopted across settings but may have potential for ambulatory care settings, such as chiropractic, physical therapy, primary care, or urgent care clinics.

Previous studies have utilized simulated cases to evaluate the utility of DDSTs in various patient populations [26, 27]. The objective of this initiative was to evaluate the utility of a CeAD DDST prior to implementation within a single Department of Veterans Affairs (VA) outpatient clinic site at VA Finger Lakes Healthcare System with the primary outcome of accuracy of identification and the secondary outcomes of inter-rater and intra-rater reliability of chiropractors’ identification of at-risk cases for CeAD that warrant advanced vascular imaging.

## Methods

This quality improvement (QI) project is reported consistent with the Standards for Quality Improvement Reporting Excellence for Simulation (SQUIRE-SIM) [28]. The VA Finger Lakes Human Research Protection Office, through the Syracuse VA Medical Center Institutional Review Board, has determined this project did not require institutional review board approval.

### Context

This QI project is a cross-sectional accuracy and inter- and intra-rater reliability assessment of the CeAD DDST. The study used CeAD-positive (CeAD+) and CeAD-negative (CeAD-) standardized clinical vignettes to simulate case information collection during history and examination in a typical clinical environment of a chiropractic clinic new patient visit.

### Setting

This project utilized LimeSurvey (Hamburg, Germany) [29], an open source, online survey platform that allows for the collection of encrypted participation response data. Access to LimeSurvey and all individual level response data were secured through LimeSurvey individual account access. All individually identifiable information obtained through the project was removed prior to data analysis.

### Participants

Seven experienced chiropractors were invited via academic and professional networks of the project team to evaluate 40 case vignettes (*n* = 10 CeAD+; *n* = 30 CeAD-) using the CeAD DDST scoring rubric and decision determination. Inclusion criteria to participate were: full, active, and unrestricted chiropractic license in a State, Territory or Commonwealth of the US, or in the District of Columbia, more than 4 years of clinical experience in an ambulatory care setting, engaged in direct patient care at least 20 h per week, no prior experience with the CeAD DDST, and fluency in English. Exclusion criteria for participants were: previous application or knowledge of the CeAD DDST, prior involvement in CeAD DDST development or validation, and prior authorship of the CeAD+ case reports or series. Participation was voluntary and participants could withdraw at any time during the study. There was no remuneration or incentive for participation.

### Interventions

#### Cervical artery dissection diagnostic decision support tool

The 4-item CeAD DDST, scored from zero to four, is determined based on (1) recent trauma or infection (yes or no), (2) acute or sudden onset (yes or no), (3) unusual head or neck pain (yes or no), and (4) presence of two or more neurological features (Table 1). A positive screen, indicated by a summative score of three or four, is deemed ‘moderate’ and ‘high’ risk for CeAD, respectively, and should be emergently referred for further vascular imaging work-up (i.e., CTA, MRA, DSA). A score of two or less is deemed ‘low’ risk for CeAD and alternate diagnoses should be considered (e.g. primary musculoskeletal pain, primary headache). In a prior study, the 4-item CeAD DDST had a sensitivity of 100%, specificity of 73.75%, “positive predictive value” of 3.81, and “negative predictive value” of 0 [16].

**Table 1:** Cervical artery dissection diagnostic decision support tool scoring criteria [16]

Criteria	Response	Score (0–4)
Acute/sudden onset	Yes	+ 1
Unusual headache or neck pain	Yes	+ 1
Trauma/infection	Yes	+ 1
Neurological features present^†^	≥ 2	+ 1

†: including but not limited to sensory loss/paresthesia, limb weakness, visual disturbance, Horner syndrome

The cervical artery dissection diagnostic decision support tool screen is positive if the score is ≥ 3 and encourages emergent referral for advanced imaging

### Measures

#### Cervical artery dissection case identification

We identified 10 CeAD+ cases in the peer-reviewed literature that described imaging-confirmed CeAD+ whose first encounter occurred in an ambulatory or outpatient setting prior to initiation of any treatment or presentation to the emergency department and appeared to be non-iatrogenic in nature. Cases were not considered if care was restricted to inpatient or emergency department presentations. Demographic data, history of the presenting illness, and physical examination findings were extracted and synthesized into narrative case vignettes using a standardized format **(**Table 2). Vignettes were reviewed and iteratively revised for clinical accuracy and consistency of presentation by senior authors (ZAC, CJF, and LRR) who had a combined 44 years of clinical experience and 32 years of clinical educator experience. If relevant case information was missing, ambiguous, or incomplete, it was left as is to reflect the reality of clinical practice.

**Table 2:** Characteristics of literature-derived cervical artery dissection positive cases

Source	Age	Sex	Imaging Modality	Evaluating Specialty	Affected Artery
Bhasvsar et al., 2024 [30]	59	Male	MRI/MRA	Ophthalmology	ICA
Buckley et al., 2023 [31]	28	Male	CTA, MRI/MRA	Urgent Care	VeA
Futch et al., 2015 [32]	30	Female	MRA	Chiropractic	VeA
Halfpap et al., 2016 [33]	51	Male	XR, CT, MRA	Physical Therapy	VeA
McCrory, 2000 [34]	27	Male	CT	Chiropractic	VeA
Michaud, 2002 [35]	42	Female	MRI/MRA	Chiropractic	VeA
Mosby & Duray, 2011 [36]	42	Female	MRA	Chiropractic	VeA
Taylor & Kerry, 2005 [37]	51	Male	US, CT, MRA	Primary Care	ICA
Wityk, 2001 [38]	46	Male	MRA	Primary Care	ICA
Swenson, 1993 [39]	31	Male	MRI, DSA	Primary Care	VeA

CT = computed tomography, CTA = computed tomography angiography, DSA = digital subtraction angiography, ICA = internal carotid artery, MRI = magnetic resonance imaging, MRA = magnetic resonance angiography, US = ultrasound, VeA = vertebral artery, XR = plain film radiography

#### Cervical artery dissection negative cases

The preliminary drafts of the simulated CeAD- cases were iteratively generated using ChatGPT GPT-5 (OpenAI, San Francisco, CA, USA) [40] utilizing clinical features of specific diagnoses commonly encountered by ambulatory chiropractic settings, informed by the 2025 National Board of Chiropractic Examiners Practice Analysis of Chiropractic [17] and chiropractic clinical practice guidelines [41]. Thirty CeAD- case vignettes exemplifying a variety of these conditions were generated (Table 3). These simulated cases ranged from 0 to 2 (“low risk”) on the CeAD DDST with a recommended decision of no need for further imaging work-up. Each vignette draft was reviewed and iteratively revised by senior authors (ZAC, CJF, LRR) for clinical accuracy and consistency of presentation.

**Table 3:** Common conditions presenting to chiropractors’ offices that were formatted as cervical artery dissection negative cases [17, 41]

Condition Name	ICD-10 Code	*N*
Cervical disc disorder with radiculopathy C5-C6	M50.122	1
Cervical disc disorder with radiculopathy C6-C7	M50.123	1
Cervical disc disorder without radiculopathy C5-C6	M50.222	1
Cervical disc disorder without radiculopathy C6-C7	M50.223	1
Cervical radiculopathy	M54.12	2
Cervicalgia	M54.2	1
Cluster headache	G44.0	1
Concussion	S06.0	1
Fibromyalgia	M79.7	1
Headache, unspecified	R51.9	2
Inflammatory arthritis	M06.4	1
Migraine with aura	G43.1	2
Migraine without aura	G43.0	1
Myalgia of auxiliary muscles, head and neck	M79.12	1
Segmental and somatic dysfunction of cervical region	M99.01	1
Spinal stenosis, cervical	M48.02	2
Spondylosis w/ myelopathy	M47.12	2
Spondylosis w/o myelopathy	M47.812	1
Sprain of ligaments of cervical spine	S13.4xxA	2
Strain of muscles, fascia, and tendons at neck level	S16.1xxA	1
Tension-type headache	G44.2	1
Thoracic outlet syndrome	G54.0	1
Torticollis	M43.6	1
Whiplash associated disorder/whiplash injury of neck	S13.4xxA	1

ICD - International Classification of Diseases

### Assessment

Raters individually assessed each clinical vignette in an independently randomized order per rater within a two-week period beginning on November 11, 2025. During the rating period, raters were asked to complete the assessment of all 40 cases in one sitting and without any external information. The assessment was created using a unidirectional logic rule to simulate realistic clinic decision making, meaning the rater rendered a final CeAD DDST score (range 0–4) and DDST-supported determination to ‘refer’ or ‘do not refer’ for advanced imaging due to “moderate” to “high” index suspicion of CeAD. They could not return to change that score or decision. Raters were given a brief introduction to the CeAD DDST and then asked to utilize the DDST to review the 40 case vignettes potentially presenting to an ambulatory clinic (see Supplementary File).

Using the CeAD DDST, raters reviewed each vignette and assigned a score and a DDST-supported clinical decision to refer or not refer for advanced imaging to further evaluate for CeAD. Raters summative score (0–4) and clinical decision to refer (‘yes’ or ‘no’) for each case vignette was captured into a standardized reporting form (LimeSurvey). The raters were not provided with any details pertaining to the make-up of the differential diagnoses presented in the case vignettes. Each rater was blinded to the proportion of CeAD + and CeAD- vignettes under review, true CeAD status of each vignette, and the responses of other raters throughout the duration of the assessment. We did not ask raters to develop a differential diagnosis or rank likelihood of potential diagnoses. The second two-week rating period commenced on January 07, 2026 with the same instructions as the first. Data were retained from raters who completed both rounds.

### Analysis

All statistical analyses were completed using RStudio Version 2026.1.1.403.11 (Posit Software, Boston, MA, USA) [42] and jamovi (Version 2.6.44.0) (The jamovi project, 2025, https://www.jamovi.org) [43].

### Primary outcome

For the primary outcome of accuracy of identification, we converted individual rater scoring to a binary threshold scoring with meeting threshold to refer (≥ 3/4 on the CeAD DDST) coded as 1 and not meeting threshold to refer (≤ 2/4 on the CeAD DDST) coded as 0. True positives (TP), true negatives (TN), false positives (FP), and false negatives (FN), were calculated (Table 4). Accuracy was defined as the sum of TP and TN divided by the total number of cases.

**Table 4:** Definitions of true positives, true negatives, false positives, and false negatives and actual counts of presented cases

	Predicted Positive	Predicted Negative
Actual Positive CeAD Case (*n* = 10)	True Positive (TP)	False Negative (FN)
Actual Negative CeAD Case (*n* = 30)	False Positive (FP)	True Negative (TN)

CeAD = cervical artery dissection, True Positive = TP: The model correctly predicts a positive outcome, True Negative = TN: The model correctly predicts a negative outcome, False Positive = FP: The model incorrectly predicts a positive outcome (Type I error), False Negative = FN: The model incorrectly predicts a negative outcome (Type II error)

### Secondary outcomes

For inter-rater reliability of rater scoring with five raters, we utilized binary threshold scoring with Fleiss’ Kappa. To determine inter-rater reliability of referral decision, we coded Yes/No responses as integer values of 1 and 0 respectively and utilized Fleiss’ Kappa. For intra-rater reliability of rater scoring, we utilized binary threshold scoring with pairwise Cohen’s Kappa. For intra-rater reliability of referral decision, we utilized pairwise Cohen’s Kappa for individual raters’ coded Yes/No scoring. All Kappa values were interpreted using the Landis and Koch criteria [44].

## Results

Of the recruited seven raters, five completed both rounds of the assessment and were included for final analysis. The two raters who did not complete both rounds either elected to end participation voluntarily due to time constraints (*n* = 1), or did not complete the initial rating round (*n* = 1). Raters had a mean age of 38.2 years (SD 11.8, range 32–59). A majority of raters were male (*n* = 3, 60%), and all raters identified as White and Not Hispanic or Latino (*n* = 5, 100%). Mean years in practice was 12.0 (SD 10.2, range 6–30) with all raters employed in an integrated care setting (*n* = 5, 100%).

### Primary outcome – accuracy of Identification in threshold scoring

No raters accurately identified all cases, with accuracy ranging between 72.5% and 95.0% (Table 5). Data tended to skew towards identification of vignettes as false positive, though false negatives were also present. The majority of raters misidentified a CeAD+ case [33] in both rounds (Round 1: *n* = 5, 100%; Round 2: *n* = 3, 60%) and a CeAD+ case [35] in Round 2 (*n* = 3, 60%). The majority of raters misidentified a CeAD- case representing a mild traumatic brain injury in both rounds (Round 1: *n* = 4, 80%; Round 2: *n* = 3, 60%), a CeAD- case representing as migraine with aura in Round 1(*n* = 3, 60%),and a CeAD- case representing an unspecified headache in Round 2 (*n* = 4, 80%).

**Table 5:** Accuracy of cervical artery dissection positive and cervical artery dissection negative case identification

	Round 1				
	TP	TN	FP	FN	Accuracy
Rater 1	9	20	10	1	72.5%
Rater 2	7	28	2	3	87.5%
Rater 3	7	28	2	3	87.5%
Rater 4	8	27	3	2	87.5%
Rater 5	9	23	7	1	80.0%

	Round 2				
	TP	TN	FP	FN	Accuracy
Rater 1	9	22s	8	1	77.5%
Rater 2	6	28	2	4	85.0%
Rater 3	9	29	1	1	95.0%
Rater 4	9	22	8	1	77.5%
Rater 5	9	27	3	1	90.0%

TP = true positive, TN = true negative, FP = false positive, FN = false negative, Accuracy = (TP + TN)/total cases (40)

### Secondary outcome- inter-rater reliability

Inter-rater reliability was assessed across five raters in both rounds (Table 6). Raters demonstrated “moderate” to “substantial” agreement beyond expected chance in Round 1 in threshold scoring of CeAD + and CeAD- cases (κ = 0.609, 95% CI 0.432–0.806), though raw percent agreement was 65%; however, raters demonstrated only “moderate” agreement beyond expected chance in Round 1 referral decision (κ = 0.567, 95% CI 0.383–0.771) with raw percent agreement at 60%. Raters demonstrated “moderate” agreement beyond expected chance in Round 2 threshold scoring of CeAD + and CeAD- cases (κ = 0.540, 95% CI 0.365–0.735) with raw percent agreement at 60% and “moderate” agreement beyond expected chance in Round 2 referral decision (κ = 0.567, 95% CI 0.377–0.761) with raw percent agreement at 63%. All inter-rater reliability scores were statistically significant (*p* < 0.001).

**Table 6:** Inter-rater reliability of rater threshold scoring and referral decision

	Kappa	Z	*p*	95% CI	% Agreement
Round 1 Threshold Score	0.609	12.2	< 0.001	0.432–0.806	65%
Round 1 Referral Decision	0.567	11.3	< 0.001	0.383–0.771	60%
Round 2 Threshold Score	0.540	10.8	< 0.001	0.365–0.735	60%
Round 2 Referral Decision	0.567	11.3	< 0.001	0.377–0.761	63%

*N* = 40, Raters = 5; Threshold Score = rater scoring of cervical artery dissection diagnostic decision support tool (score range: 0–4; ≥3 refer for additional imaging work-up); Referral Decision = rater referral decision (Yes/No)

### Secondary outcome – intra-rater reliability

Intra-rater reliability among raters ranged from “moderate” agreement to “almost perfect” agreement beyond expected chance (range 0.457–0.931) in both threshold scoring and referral decision (Table 7). Raw percent agreement among raters ranged from 75% to 98%. All intra-rater reliability scores were statistically significant (*p* ≤ 0.003).

**Table 7:** Intra-rater reliability of rater threshold scoring and referral decision

		Round 1 and Round 2				
		Kappa	Z	P	95% CI	% Agreement
Threshold Score	Rater 1	0.698	4.44	< 0.001	0.476–0.920	85%
	Rater 2	0.627	3.98	< 0.001	0.330–0.924	88%
	Rater 3	0.655	4.15	< 0.001	0.378–0.932	88%
	Rater 4	0.571	3.81	< 0.001	0.320–0.822	80%
	Rater 5	0.457	2.96	0.003	0.176–0.737	75%
Referral Decision	Rater 1	0.698	4.44	< 0.002	0.476–0.920	85%
	Rater 2	0.793	5.03	< 0.001	0.570-1.000	93%
	Rater 3	0.931	5.90	< 0.001	0.798-1.000	98%
	Rater 4	0.571	3.81	< 0.001	0.320–0.822	80%
	Rater 5	0.457	2.96	0.003	0.176–0.737	75%

*N* = 40, Raters = 5; Threshold Score = rater scoring of cervical artery dissection diagnostic decision support tool (0–4); Referral Decision = rater referral decision (Yes/No)

## Discussion

This QI project assessed the accuracy of identification, inter-rater reliability, and intra-rater reliability of the CeAD DDST using CeAD+ cases reported in the literature and simulated cases reflective of CeAD- conditions commonly presenting to ambulatory settings. Accurate identification of CeAD + and CeAD- cases varied among raters, ranging from 72.5% to 95.0%. False negatives, though low, were still present in our data compared to Thomas et al. [16] who reported 100% sensitivity with an emergency department cohort. There is a discrepancy between the false negatives present in our data and lack of false negatives in the data used to validate the CeAD DDST. In contrast to Thomas et al. [14, 16], who exclusively utilized real patient cases, we used simulated cases in the setting of this process improvement project and therefore may have limited the full clinical picture for raters. We do not recommend implementation of the CeAD DDST in the ambulatory setting at this time without further refinement of the DDST to avoid compromising patient safety given the urgent nature of CeAD.

For DDSTs to be considered of utility, it is generally considered that a higher inter-rater reliability score is more useful in the clinical setting to limit the amount of potential missed cases, though there is no agreement upon what value should be threshold. It has been suggested that absolute agreement among raters should exceed 80% or kappa ≥ 0.60 for clinical testing, with higher values more desirable in high-stakes contexts [45]. Current data fall short of this benchmark, leading to limitations in immediate implementation within the ambulatory setting. It may be possible to meet these suggested benchmarks with a combination of iterative CeAD DDST revision to consider inclusion of established environmental exposures, genetic or congenital predispositions, and anatomic considerations, clinician training, and end-user feedback [1].

Our target clinic environment contrasts with the CeAD DDST validation population, an emergency department cohort [16]. Signs and symptoms in patients who present to an emergency department for evaluation may suggest more concerning clinical features. This may relate to a greater likelihood of neurological symptoms consistent with later stage CeAD pathology progressing to ischemic events compromising neurological structures, rather than absent or transient neurological symptoms that may be present in earlier CeAD manifestations. Thomas et al. noted that approximately 25% of the cases included in the validation cohort had previously presented to an ambulatory care setting and were misdiagnosed [16]. This is consistent with the evolving nature of CeAD, in which prodromal symptoms like neck and/or head pain prompt an encounter with an ambulatory care provider, though the clinical findings may not have progressed to a point of meeting threshold for this DDST or a patient seeking emergent care [5, 9, 13]. Further, this may explain why CeAD+ cases from ambulatory settings failed to reach a threshold score of three with the CeAD DDST, assuming case presentation is earlier in the condition evolution.

Ambulatory care providers are optimally positioned to evaluate and refer for emergency care upon the suspicion of CeAD prior to or at the early emergence of associated neurological symptoms; however, there is a concern of reduced sensitivity of the CeAD DDST in early disease, as the patients most likely to be helped by early intervention may be at a stage of pathology where their score does not meet threshold on the DDST and are triaged as low risk [9, 13]. The ability for a screening tool to be effective relies on its sensitivity, though a high sensitivity alone does not necessarily reflect clinical utility in decision-making for individual patients and should not replace clinical reasoning performed by the clinician [46]. Positive predictive values and negative predictive values are more useful as they reflect the *person* compared to the reference standard, though all four values are often reported together to provide greater context [46]. In review of Thomas et al., it appears they reported a “positive predictive value” of 3.81 and a “negative predictive value” of 0, with both values being illogical compared to the definition of predictive values and the results of their data [16]. In reviewing their data, these values likely represent likelihood ratios, rather than predictive values. In light of this, further evaluation of this tool is required to accurately display utility as there is conflicting information, and the results of this QI project do not support the translation into ambulatory clinical practice at this time.

Frameworks regarding cervical spine red flags and assessing for serious vascular pathologies have been previously developed, which may serve as a training companion for the current DDST [24, 47]. In this test implementation of the CeAD DDST, we elected specifically not to train raters on the use of the tool beyond the provided scoring, which mimics real-world implementation of the DDST for end users. The variability among raters through natural use of the DDST demonstrates potential ambiguity in the exact definitions of the criteria which comprise the tool. This is further supported by the high degree of false positives, consistent with Thomas et al., [16] demonstrating overlap with cases such as migraine and traumatic brain injury. Our findings suggest participants demonstrated potentially poor discrimination between migraine, traumatic brain injury, and CeAD which may be a preliminary signal of educational deficiency and may be a target for curricular enhancement at the pre-professional and continuing education levels. While we anticipate the presence of false positives for a successful highly sensitive tool and desire to refer all cases of true CeAD, a high degree of false positives may negatively impact downstream healthcare costs, resource utilization, and patient burden, resulting in undue financial and psychological stress [48]. Further development of this DDST, or consideration of use in conjunction with other frameworks specific to vascular pathology detection, may provide greater utility for use in the clinical setting and reduce unnecessary false positive and false negative screening.

The utility of the CeAD DDST is only secondary to its usability, and the input from the end user likely will help limit any confusion and decisional uncertainty, improving outcomes for patients and providers. Given the output variability of the CeAD DDST and ambiguity surrounding the criteria, future considerations should take into account the end user’s vantage point, barriers to, and facilitators of incorporating the tool in clinic workflows for progressive, iterative refinement. The current project demonstrated foundational usability and performance data that may inform further revision of the tool. Thomas et al. recommended incorporating the SNNOOP10 criteria [49], a screening mnemonic to identify red flags in headache patients, and/or inclusion of the age criterion, which was not evaluated in this study [16]. It may be useful to align the CeAD DDST with already established red flag screening infrastructure to improve end user operability in clinic workflows [24]. The backbone of the tool’s functionality is enhancing operational definitions for each criterion to limit confusion or ambiguity among clinicians implementing the tool, creating greater efficiency and inter-rater reliability.

### Strengths and limitations

There are several limitations to consider. We conducted a preliminary evaluation of the CeAD DDST in a simulated ambulatory care setting using case vignettes and did not implement the tool into real clinical workflows with real patients where a care decision may impact a patient’s health and life. This simulated context may have impacted clinician decision making, since no patients could be harmed based on the decision made using the tool. Real-world CeAD+ cases provide a reference standard for determining the utility of this tool in the ambulatory care setting without the potential for patient harm during the duration of the study. Generative artificial intelligence cases, however, are not a substitute for evaluating this tool in the clinical setting. As this is a preliminary investigation, utilization of ChatGPT, informed by commonly seen conditions and senior clinician experience, provides an ethical, straightforward way of creating control cases that can plausibly reflect real clinical scenarios [50]. It also creates substantiation for further study in ambulatory care clinics once there is confidence in utility of the tool and appropriate case discrimination. The heterogeneity in case detail among published CeAD+ case reports creates difficulties in the presentation of complete clinical information for raters but may simulate barriers found with patients in clinical practice.

Future studies should consider use of real clinical vignettes in CeAD + and CeAD- cases that include pertinent medial history components that may allow a clinician to consider the individual context of each clinical vignette. We did not prompt the raters to consider or rank differential diagnoses which may have led to different decision making and DDST scoring and may be an important instructional detail for future DDST iterations. The current pool of raters were not representative of all end-users, and future DDST evaluations should encompass a larger, more representative multi-disciplinary clinician sample. Sensitivity and specificity were not directly assessed as the purpose of this project was to evaluate the utility of the DDST, which has already established sensitivity and specificity. We did not consider qualitative investigation for this project which may have been valuable to discern usability of the DDST, barriers to and facilitators of accurate DDST use, and feedback on education and training for the DDST, though the current project lays the groundwork for future implementation of qualitative work.

## Conclusions

This preliminary analysis of a validated CeAD DDST demonstrates that accuracy, inter-rater reliability, and intra-rater reliability of chiropractors utilizing the tool in a simulated ambulatory setting may not provide clinical utility at this time. This DDST may be useful in an emergency department setting given it was developed with this data, but implementation into the clinical workflow in an ambulatory setting is not currently recommended for the outpatient ambulatory clinic given the wide variability in end user implementation and presence of false negatives. Further refinement and testing should be completed prior to introduction into the clinical workflow.

## Supplementary Information

Below is the link to the electronic supplementary material.

Supplementary Material 1

## Data Availability

The datasets used and/or analyzed during the current study are available from the corresponding author on reasonable request.

## Declarations

### Disclaimers

The contents of this study are solely the responsibility of the authors and do not represent the official views of affiliate institutions, the U.S. Department of Veteran’s Affairs, or the United States Government.

## Abbreviations

CeAD: Cervical artery dissection
CeAD-: Cervical artery dissection negative
CeAD+: Cervical artery dissection positive
CT: Computed Tomography
CTA: Computed tomography angiography
DDST: Diagnostic decision support tool
DSA: Digital subtraction angiography
FN: False negative
FP: False positive
ICA: Internal carotid artery
ICD: International Classification of Diseases
MRA: Magnetic resonance angiography
MRI: Magnetic resonance imaging
QI: Quality improvement
SD: Standard deviation
SQUIRE-SIM: Standards for Quality Improvement Reporting Excellence for Simulation
TN: True negative
TP: True positive
US: Ultrasound
VA: Department of Veterans Affairs
VeA: Vertebral artery
XR: Plain film radiography
