# Supplementary material for: Preliminary accuracy, inter- and intra-rater reliability of a 4-point cervical artery dissection diagnostic decision support tool in an ambulatory setting using real and simulated cases: a quality improvement project
Source: Chiropr Man Therap. 2026 Oct 11;34:59. doi: 10.1186/s12998-026-00679-3 (PMC13654208; doi:10.1186/s12998-026-00679-3)
Supplement: Supplementary file 1 [file 12998_2026_679_MOESM1_ESM.docx]

Table of Contents

[Rater Instructions	2](#_Toc233202437)

[CeAD+ Cases	5](#_Toc233202438)

[CeAD- Cases	9](#_Toc233202439)

Rater Instructions

Thank you for your participation in this quality improvement study. The link you have received is unique and individualized for one time use. You will be utilizing a validated cervical artery dissection diagnostic decision support tool (CeAD DDST) evaluate 40 cases of neck pain and headache presenting to a chiropractor’s office. There are 4 criteria used for this tool: acute/sudden onset of pain, unusual/unfamiliar pain, presence of ≥2 neurological features, and recent trauma/infection. Each criterion present is 1 point, and the score is summative of all criteria. The tool is shown in the table below. This table will be available to you for the duration of the study.

| **Criteria** | **Category** | **Score (0-4)** |
| --- | --- | --- |
| Acute/sudden onset | Yes | +1 |
| Unusual headache or neck pain | Yes | +1 |
| Trauma/infection | Yes | +1 |
| Neurological features | ≥2 | +1 |

The CeAD DDST is scored on a scale from 0-4, with a score of ≥3/4 denoting that a patient is at “moderate” to “high” risk for CeAD and should be referred for further imaging work-up (i.e., neck magnetic resonance angiogram…). Scoring is shown below, and will be available to you for the duration of the study:

| Score |  | Action |
| --- | --- | --- |
| ≤2/4 | Low risk for CeAD | Consider alternate diagnosis |
| 3/4 | Moderate risk for CeAD | Emergently refer for imaging and monitor |
| 4/4 | High risk for CeAD | Emergently refer for imaging and monitor |

For example, a case of sudden and new neck pain in the setting of recent motor vehicle collision, without unusual pain or neurological features would score a 2/4. The interpretation of this score is considered ‘low risk for CeAD’, and an alternate diagnosis should be considered, warranting no referral for further diagnostic work-up.

Each case vignette will be presented to you on a single page one vignette at a time, and the order of vignette presentations has been randomized. Please read the entirety of the case. After reading the case, utilize the CeAD DDST provided and score the case based on the features present. You will select a score from the drop-down box, 0 to 4, provided. Based on your clinical judgment and informed by the CeAD DDST score, you will render a clinical referral decision (i.e., to refer or to not refer). Once you have rendered decisions and select ‘next vignette’ and transitioned to the next page, you will be unable to return to the previous vignette.

You will have unlimited time to complete your vignette review, CeAD DDST scoring, and clinical referral decision. We ask that all 40 cases be evaluated in one sitting – you will not be able to close and return to the form at a later time. Please do not use any resources external to what is provided to you in the form itself and complete this independently. Accuracy of your clinical referral decision, CeAD DDST scoring, and the definitive diagnostic impressions will NOT be shared with you. Please begin when you are ready.

CeAD+ Cases

| **CaseID** | **Case Reference** | **Text** |
| --- | --- | --- |
| posCase_1 | Bhavsar et al 2024^1^ | A 59-year-old male patient presents with a left-sided headache over the past three days. Over that time, he noticed that his left eyelid was drooping when he looked in the mirror. He also became aware of difficulty swallowing and moving his tongue. Nothing he has done improved or worsened the headache. He is an active hiker and often goes to the gym, though he denies any recent trauma. Past medical history is unremarkable and he takes no medications. Visual inspection showed mild drooping of the left upper eyelid, as well as asymmetric pupil sizes, with the left pupil measuring 2 millimeters compared to the right at 4 millimeters. Cranial nerve examination showed left deviation of the tongue with protrusion, though the remainder of the examination was normal. |
| posCase_2 | Buckely et al 2023^2^ | A 28-year-old male presents with sudden-onset right-sided neck pain with radiation to the right frontal region of his head. This began two weeks ago during a CrossFit boot camp workout. When it started, he noted that he felt “confused.” Both of his arms went numb and he had difficulty walking. He denied any recent trauma. When he presents now, he does have some difficulty speaking and the right side of his face appears to be drooping compared to the left. His past medical history is unremarkable. Cranial nerve examination shows the inability to abduct his right eye on conjugate gaze testing and static constriction of the right pupil. Coordination testing revealed right-sided dysmetria. The remainder of the examination was unremarkable. |
| posCase_3 | Futch et al 2015^3^ | A 30-year-old female presents with right-sided neck pain in the suboccipital region with sudden, though transient, blurriness of her right eye. Two weeks ago, she had an episode of severe left-sided neck pain and headache without any trauma, described as “like nothing I’ve ever experienced before,” after which the right-sided neck pain began. Three days ago, she visited the emergency department because she lost her left-sided peripheral vision and felt “numbness” in her left eyelid, which the emergency department diagnosed as an ocular migraine. She does have a history of ocular migraines without prodrome which she states are related to her menstrual cycle, but she has not experienced visual changes with them. She also gave birth 2.5 months ago. She also notes a family medical history of spontaneous ascending thoracic aortic aneurysm. Neurological examination showed no sensory or motor changes in the upper and lower extremities, normal gait, and normal balance. |
| posCase_5 | Halfpap et al 2016^4^ | A 51-year-old male presents with a five-day history of persistent neck pain following a fall in shallow water while surfing, with intensity ranging from 2-7/10. Neck pain is present over the mid to lower cervical paraspinal region. When he fell, he hit the left side of his head, forcing it into right lateral flexion and compressed his head down onto his neck. Immediately, he felt pain into his right upper extremity that quickly went away. He denies losing consciousness, headache, dizziness, vertigo, or paresthesia. He resumed swimming a day later. On examination, he is alert and in no acute distress. Global cervical range of motion was mildly limited. |
| posCase_12 | McCrory 2000^5^ | A 27-year-old male presents with “deep pain” in his neck and “pins and needles” down his right arm. Pain began two days ago after being tackled in a rugby match, where he was struck on the right side of the neck. He initially declined medical treatment. Yesterday, he notes difficulty walking, as well as slurring his speech. Neurologic examination does not currently show this, and is otherwise normal. |
| posCase_13 | Michaud 2002^6^ | A 42-year-old female presents with unilateral left-sided suboccipital headache over the past 2 weeks. The headache began after she was lifting sheets of drywall on top of her car. She remembers "twisting" her neck as she was lifting and immediately having an intense, deep pressure that persisted without change. She does have a past medical history of occasional tension type headaches, which she associates with poor posture at her workplace. She repeatedly emphasizes that this headache is unlike any headache she has ever experienced. Neurologic examination shows pupils equal, round, and reactive to light, intact and equal bilateral upper and lower reflexes, vibration sense, point localization, and 2-point discrimination. Sensory, motor, and coordination are intact and equal bilaterally without deficit. Babinski sign is plantar and no ankle clonus is appreciated. Cranial nerve examination is normal. Cervical compression testing displays slight discomfort, as well as slight restrictions in bilateral cervical rotation and flexion. |
| posCase_14 | Mosby & Duray 2011^7^ | A 42-year-old female presents with complaint of left-sided neck and shoulder pain, described as burning and sharp. She states that she regularly self-manipulates her neck to reduce neck pain several times a day. On physical examination, there is “burning” on the left side of C7 with palpation, myospasms of right-sided thoracic paraspinal muscles, and myospasms on the left-sided lumbar paraspinal muscles. Cervical compression and shoulder depression tests cause pain in the left lower cervical region. Further pressing into history reveals a persistent headache that began twelve days prior. Her neck pain ascended into the temporal region on the left side, being described as stabbing and worse than she has ever experienced. She rated her pain 10/10 on the numeric pain rating scale. She also had episodes of nausea and vomiting. Headaches would occur for three hours per day. She denied any tobacco or oral contraceptive use, and endorsed social alcohol consumption. |
| posCase_15 | Taylor and Kerry 2005^8^ | A 51-year-old male presents with left-sided mid to upper cervical pain with aching in the left temporomandibular joint and mandible region. This began after he attended a pre-Christmas dinner party when he had a sudden urge to sneeze. He turned his head fully to the left and sneezed several times. He "jarred" his neck at the time and gradually became aware of the pain. He completed his meal with some discomfort. The next day, symptoms persisted as left suboccipital region pain and difficulty turning his head to the left. He also had a left-sided headache that affected his left frontal region and eye. Pain intensity rated 6/10 and continued throughout the day. He used NSAIDs with relief. Over the next 48 hours his pain remained constant. While driving to work 48 hours later, he became aware of drooping of his left eyelid and constriction of the pupil on the left side. |
| posCase_17 | Wityk 2001^9^ | A 46-year-old male presents with an acute 2-day history of headache centered on the right temple after working out on an exercise treadmill. The headache persisted for the entirety of the 2 days prior to presentation. He also feels like he lost his appetite and is feeling a bit ill, stating flulike symptoms. He was driving home from work when he realized that he sideswiped a cement bridge. He felt like he could not control his car. Without realizing it, he straddled a guardrail and went over it, missing his exit. The police came and on visual inspection he had different sized pupils and asymmetric grip strength. |
| posCase_18 | Swenson 1993^10^ | A 31-year-old male presents with a left-sided occipital headache that began 3 weeks prior. It began as intermittent but became constant. Two days ago, he noted sharp, left-sided neck pain in the suboccipital region. Today, he developed sudden onset of vertigo with nausea and vomiting. He has a past medical history of heroin, cocaine, and marijuana abuse. Physical examination showed horizontal nystagmus and mild dysarthria. Pupils were equal, round, and reactive to light. His right eye was exotropic. He is currently unable to adduct the right and left eyes. Muscle strength is intact in all extremities. Deep tendon reflexes are brisk with Babinski sign present bilaterally. He also has clonus in the left ankle. Sensory examination shows decreased pinprick and temperature sense in the right upper and lower extremity and the left side of his face. |

CeAD- Cases

| **CaseID** | **ICD10- Primary Condition** | **Case text** |
| --- | --- | --- |
| negCase_1 | S13.4xxA — Sprain of ligaments of cervical spine | A 35-year-old male presents with neck pain that began after lifting a heavy box awkwardly at work two days ago. He recalls feeling a sudden “twinge” at the base of his neck, which was followed by “stiffness” that worsened overnight. The pain is localized to the midline lower cervical region and occasionally radiates toward the right shoulder blade, but he denies arm numbness, weakness, or tingling. He describes the discomfort as a constant dull ache, rated 5/10, aggravated by turning his head to the right or looking up. His medical history is unremarkable except for occasional seasonal sinus infections. On examination, vital signs are within normal limits, and he is alert and oriented. Active cervical rotation and extension are moderately limited, with reproduction of their pain at end ranges. Palpation reveals tenderness over the spinous processes and paraspinal musculature of the lower cervical spine. Upper extremity strength, reflexes, and sensation are intact without deficit. |
| negCase_2 | S16.1xxA — Strain of muscles, fascia, and tendons at neck level | A 29-year-old female reports the onset of neck pain after spending several hours painting the ceiling of her new apartment three days ago. She recalls gradually developing a pulling sensation along the back of her neck, which later became a persistent ache. The discomfort is worse at the end of the day and when she tries to look upward for extended periods. She denies headache, dizziness, numbness, or tingling in her arms. She has no significant past medical history but does mention her regular yoga practice. On physical examination, a mild forward head positioning posture is appreciated. Palpation reveals tenderness and taut bands in the upper trapezius and cervical paraspinal muscles. Cervical active range of motion is mildly restricted in extension and left rotation, both of which reproduce her symptoms. Neurological screening, including reflexes, sensation, and motor strength in the upper extremities, is intact without deficit. Vital signs are stable and no abnormal eye movements or balance changes are noted. |
| negCase_3 | M54.2 — Cervicalgia | A 47-year-old woman presents with a complaint of neck discomfort that began about eight weeks ago without a clear trigger. She describes a steady, nagging ache in the left side of her neck that sometimes spreads toward the base of her skull. She has been working long hours at a computer recently and attributes some of her stiffness to poor posture. She denies dizziness, visual changes, or weakness in her arms, though she mentions occasional tingling in her left hand at night that resolves when she shakes it out. Her past history includes mild hypertension, managed with medication. On examination, she appears mildly uncomfortable when turning her head to the left. No obvious deformity or swelling is noted. Grip strength is intact and reflexes in the upper limbs are symmetric. Vital signs are stable. |
| negCase_4 | M99.01 — Segmental and somatic dysfunction of cervical region | A 38-year-old male presents with neck stiffness and intermittent pain over the past five days. He describes a dull ache in the right posterior neck with occasional sharp discomfort when quickly rotating his head left or right. He reports spending several hours each evening working on his laptop while sitting on the couch, which he feels may be contributing to his neck stiffness. He denies headache, dizziness, or visual changes. Past medical history is unremarkable aside from mild seasonal allergies. On examination, his head carriage appears slightly forward, and cervical rotation is more limited to the right than left. Palpation reveals localized tenderness and increased muscle tension along the right cervical paraspinals. Upper extremity strength and sensation are intact without deficit. No abnormal eye movements are observed. Vital signs are within normal limits. |
| negCase_5 | M79.12 — Myalgia of auxiliary muscles, head and neck | A 33-year-old female presents with aching discomfort along the left side of her neck and upper shoulder that has persisted for about six weeks. She recalls no injury but mentions that she recently began carrying a heavy shoulder bag while commuting. The pain is described as a dull, constant ache that worsens by the end of the day and occasionally radiates toward the jawline. Her past medical history is notable for mild asthma. On examination, there is visible tightness in the left upper trapezius with tenderness to palpation. Cervical motion is generally preserved, though lateral flexion to the right provokes discomfort. Neurologic screening of the upper extremities demonstrates intact reflexes and normal motor function. |
| negCase_6 | S13.4xxA — Whiplash associated disorder / whiplash injury of neck | A 26-year-old female reports neck pain and stiffness beginning the morning after being involved in a rear-end motor vehicle collision two days ago. She describes a deep ache across the back of her neck with sharp pain when turning her head quickly. She denies loss of consciousness at the time of the accident and has had no dizziness, visual changes, or limb weakness since. Her past history includes major depressive disorder and appendectomy but is otherwise unremarkable. On examination, she appears guarded in her movements and avoids rapid head rotation. Cervical range of motion is moderately reduced in all planes due to pain, most notably in extension. Tenderness is noted over the cervical paraspinals bilaterally. Reflexes and upper limb strength are symmetric. Sensation is intact to light touch throughout the arms. Vital signs are within normal limits. |
| negCase_7 | R51.9 — Headache, unspecified | A 41-year-old male presents with intermittent headaches over the past four days. The pain began suddenly while he was at work, described as a pressure-like ache across the right side of his head that occasionally radiates toward the temple. He states the discomfort is unlike the mild tension headaches he has had in the past, rating it 6/10 at its worst. He denies nausea, vomiting, or sensitivity to light.. Past medical history is notable for seasonal allergies and occasional sinus congestion, though he denies recent upper respiratory symptoms. On examination, he appears uncomfortable but alert and oriented. Palpation over the cervical paraspinal region does not reproduce symptoms. Cervical range of motion is full. Neurologic screening shows intact strength and reflexes in the upper extremities. Cranial nerves are grossly normal, though he reports slight eye strain during prolonged right gaze. |
| negCase_8 | G43.0 — Migraine without aura | A 32-year-old female presents with a three-day history of throbbing headaches located on the left side of her head. The pain builds gradually, lasting several hours at a time, and worsens with bright light and loud noise. She reports mild nausea but no vomiting. She denies visual changes, dizziness, or limb weakness. She has experienced similar headaches sporadically since her late teens, though she notes this episode feels more prolonged than usual. Her mother also has a history of recurrent headaches. On examination, she appears fatigued and prefers to keep the lights dimmed. Palpation over the scalp and cervical paraspinal muscles does not change her symptoms. Cervical motion is unremarkable. Neurological exam shows normal coordination, strength, and reflexes throughout. Cranial nerves are intact. |
| negCase_9 | G43.1 — Migraine with aura | A 27-year-old male presents with recurrent headaches over the past year, the most recent beginning two days ago. He describes a pulsating pain on the right side of his head, typically preceded by visual disturbances that he refers to as “shimmering zig-zags” lasting about 20 minutes. This episode followed the same pattern. The headache builds to a moderate intensity and is accompanied by sensitivity to light and sound. He denies nausea, vomiting, or limb weakness. He reports that the pain is relieved somewhat by resting in a quiet, dark room. His past medical history is unremarkable, and he does not take any regular medications. On examination, he appears uncomfortable but in no acute distress. Cervical motion is full without reproduction of pain. Palpation of the suboccipital muscles elicits mild tenderness but does not change the headache. Neurological screening is grossly normal, though he briefly reports blurred vision on sustained right gaze. Cranial nerves otherwise appear intact. |
| negCase_10 | G44.0 — Cluster headache | A 36-year-old male presents with excruciating left-sided headaches that have woken him from sleep every night for the past week. He describes the pain as “like someone is stabbing a hot poker behind my eye,” lasting around 40 minutes before easing. During these attacks, his left eye waters and his nose becomes congested, forcing him to pace around the room because lying down worsens the discomfort. He recalls a similar episode two years ago that resolved after several weeks on its own. He denies nausea, vomiting, or limb weakness. He works as a bartender and notes the pain seems worse on nights after drinking even a small amount of alcohol. On examination, his left eye appears injected with mild ptosis, and there is tearing at the medial canthus. Pupils are reactive and equal. Extraocular movements are intact. Facial sensation and motor function are preserved. Reflexes in the upper extremities are symmetric and normal. |
| negCase_11 | G44.2 — Tension-type headache | A 44-year-old female reports a two-week history of a steady, band-like pressure across her forehead and temples that often worsens toward the end of her workday. She works long hours at a desk and attributes some of her discomfort to stress and posture. She describes the pain as a tight, squeezing sensation rather than sharp, with intensity ranging from 3–6/10. She denies nausea, vomiting, or visual changes, but mentions mild sensitivity to sound when the headache is at its worst. She has a medical history that includes generalized anxiety disorder, bipolar disorder, and mild seasonal allergies. On examination, her posture demonstrates forward head positioning and rounded shoulders. Palpation reveals tenderness in the suboccipital region and tightness of the upper trapezius bilaterally. Cervical range of motion is full, though she reports pulling discomfort with end-range flexion. Neurological exam shows symmetric strength and reflexes in the upper limbs, though a unilateral positive Hoffman’s sign on the left. |
| negCase_12 | M43.6 — Torticollis | A 22-year-old male presents with sudden onset of painful neck stiffness that began after waking up in the morning two days ago. He reports his head has been “stuck” tilted slightly to the right with difficulty turning it back to neutral. The pain is described as sharp at the base of the neck and dull along the right side, worsening with attempts to rotate left. He denies headache, dizziness, numbness, or tingling in the arms. He has no prior history of neck problems. Medical history is significant for type I diabetes. On examination, his head is held in right lateral flexion with mild rotation. Attempts at active correction are limited and painful. Palpation shows tightness and tenderness in the right sternocleidomastoid and upper trapezius. Neurologic screening demonstrates intact strength and reflexes in the upper extremities. |
| negCase_13 | M50.122 — Cervical disc disorder with radiculopathy, C5–C6 | A 49-year-old male presents with a seven-week history of neck pain radiating into his right shoulder and down the arm. He describes the discomfort as a deep ache with intermittent sharp, shooting pains that extend toward the thumb and index finger. The pain is aggravated by prolonged computer work and driving, and partially relieved when he supports his arm on the armrest. He denies dizziness or visual changes but notes occasional tingling in his right hand, especially at night. His past medical history is notable for hyperlipidemia and L4-L5 discectomy. On examination, cervical range of motion is reduced, particularly with extension and right rotation, both reproducing symptoms. Spurling’s maneuver increases pain radiating into the right arm. Strength testing shows mild weakness with elbow flexion on the right compared to the left. Reflexes are diminished at the right biceps, while triceps and brachioradialis reflexes remain symmetric. Sensation is decreased along the lateral forearm. |
| negCase_14 | M50.123 — Cervical disc disorder with radiculopathy, C6–C7 | A 52-year-old female presents with neck pain and intermittent shooting discomfort down her left arm that began insidiously over a month ago. She describes a burning sensation that radiates from the lower neck into the triceps region and sometimes toward the middle finger. She denies dizziness, headache, or visual disturbances. She notes difficulty gripping a gallon of milk with her left hand over the past week, though she is unsure if it is related to pain or weakness. Her medical history includes status-post total right hip arthroplasty, well-controlled hypothyroidism and past cholecystectomy. On examination, cervical extension and left rotation are limited and provoke radiating pain into the arm. Strength testing reveals mild weakness in wrist flexion on the left compared to the right. Reflexes are reduced at the left triceps, while biceps and brachioradialis reflexes are symmetric. Sensation is diminished over the posterior aspect of the left forearm. |
| negCase_15 | M50.222 — Other cervical disc displacement, mid-cervical region | A 45-year-old male presents with a two-week history of neck stiffness and intermittent pain radiating between the shoulder blades. He reports the pain worsens after long drives and when turning his head quickly, occasionally sending a sharp jolt into the mid-back. He denies arm numbness, tingling, or weakness. His past medical history is notable for mild gastroesophageal reflux disease and tinnitus. On examination, posture shows a slight forward head position with limited cervical rotation bilaterally, more pronounced to the left. Palpation elicits tenderness along the mid-cervical paraspinal muscles and bilateral rhomboids. Spurling’s maneuver does not reproduce symptoms. Strength and reflexes in the upper extremities are normal. Sensation is intact. While performing cervical flexion, he briefly reports a transient pulling sensation radiating toward the left scapular region. |
| negCase_16 | M50.223 — Other cervical disc displacement, cervicothoracic region | A 56-year-old female presents with persistent neck and upper back discomfort that has gradually worsened over the past two months. She describes a dull ache at the base of her neck radiating between the shoulder blades, occasionally sharp with overhead reaching or prolonged reading in bed. She denies headaches, dizziness, or limb numbness, though she mentions her left arm has felt “tired” after household chores, but she is not dropping things. Past medical history includes hypertension and osteoarthritis of both knees. On examination, cervical flexion is full, but extension produces discomfort radiating toward the upper thoracic region. Palpation reveals tenderness along the cervicothoracic junction with palpable tightness in the paraspinal musculature. Motor strength in the upper extremities is symmetric, and reflexes are normal without deficit. Sensation is intact. During testing of shoulder abduction, she hesitates, noting mild discomfort radiating into the upper thoracic spine, though no weakness is observed. |
| negCase_17 | M54.12 — Radiculopathy, cervical region | A 43-year-old male presents with a ten-day history of neck pain radiating into his right arm. He describes a sharp, electric-like discomfort that travels from the side of his neck into the lateral arm and occasionally into the hand. He reports intermittent tingling in his index and middle fingers, worse when he tilts his head backward. He denies dizziness, visual changes, or gait problems. His past history is otherwise unremarkable, though he has had similar episodes of this pain twice in the past year. On examination, cervical extension reproduces radiating symptoms down the arm. Spurling’s maneuver on the right side increases his discomfort. Motor testing shows mild weakness in wrist extension compared to the left. Reflexes are diminished at the right triceps, while biceps reflexes remain symmetric. Sensation is decreased over the dorsum of the right hand. |
| negCase_18 | M47.812 — Spondylosis without myelopathy or radiculopathy, cervical region | A 61-year-old female presents with chronic neck stiffness and intermittent aching pain that has been gradually worsening over the past six months. She describes the discomfort as a dull, grinding sensation, particularly noticeable when turning her head while driving. She denies arm weakness, numbness, or tingling, though she does report occasional crepitus with movement. Past history includes hypercholesterolemia and mild osteopenia. On examination, cervical range of motion is reduced, especially with extension and rotation. Palpation reveals tenderness over the lower cervical spinous processes and paraspinals. Neurological screening shows intact strength, reflexes, and sensation in the upper extremities. While performing tandem gait, she demonstrates a slight wobble, which she attributes to being “out of practice” walking in a straight line, but she recovers without assistance. |
| negCase_19 | M47.12 — Other spondylosis with radiculopathy, cervical region | A 58-year-old male presents with a four-year history of neck pain that has recently become more bothersome, with new onset shooting discomfort down his left arm over the past three days. He reports the pain as a dull ache in the lower neck that intermittently radiates into the shoulder and forearm, sometimes associated with tingling in the third digit. He denies headache, dizziness, or visual changes. His medical history is significant for well-managed type 2 diabetes and congenital lumbar spinal stenosis. On examination, cervical extension and left rotation provoke radiating symptoms. Strength testing reveals mild weakness in left wrist extension compared to the right. Deep tendon reflexes show a slight reduction at the left triceps. Sensation is diminished over the dorsal forearm. Palpation demonstrates stiffness and tenderness over the lower cervical spine. |
| negCase_20 | M47.12 — Other spondylosis with myelopathy, cervical region | A 64-year-old female presents with gradually worsening neck stiffness and vague unsteadiness when walking over the past two months. She describes intermittent aching in the back of her neck, sometimes radiating between the shoulder blades. She denies arm pain but notes occasional tingling in both hands, especially when holding her phone for long periods. Her medical history includes uncontrolled hypertension and tobacco use. On examination, cervical extension is limited and reproduces a sense of pressure in the neck. Motor testing shows mild weakness in grip strength bilaterally, otherwise upper extremity strength is intact. Reflexes are brisk in the upper extremities, and Hoffman’s sign is present on the right. Sensation is intact to light touch but diminished slightly for vibration at the fingertips. Tandem gait is unsteady, and she requires a step to regain balance. Cranial nerves appear normal. |
| negCase_21 | G54.0 — Brachial plexus disorders | A 40-year-old male presents with persistent right shoulder and upper arm pain following a fall from his bicycle five weeks ago. He recalls landing awkwardly on his side with his arm stretched overhead. He does not recall hitting his head and did not lose consciousness. Since then, he has experienced a deep ache in the shoulder with intermittent sharp pains radiating down the lateral arm. He also describes occasional tingling in the thumb and index finger, especially after prolonged activity. He does state that he had similar symptoms prior to the fall, though they are worse now. He denies dizziness, headache, or visual disturbances. Medical history remarkable for exertional asthma and basal cell carcinoma of the scalp. On examination, there is visible guarding of the right shoulder with reduced active abduction due to pain. Strength testing reveals mild weakness in elbow flexion and shoulder abduction on the right compared to the left. Reflexes are symmetric, though biceps reflex is slightly diminished on the right. Sensation is decreased over the lateral forearm. Palpation of the supraclavicular region elicits localized tenderness without swelling. |
| negCase_22 | M79.7 — Fibromyalgia | A 46-year-old female presents with widespread muscle aches and persistent fatigue that have been ongoing for several years. She describes the pain as a diffuse, dull ache affecting the neck, jaw, shoulders, and upper back, worse in the morning and after long days at her office job. She also reports difficulty sleeping and occasional headaches, which she attributes to stress. She denies numbness, tingling, or focal weakness. Her medical history is notable for TMJ disorder, sleep apnea managed with CPAP, mild depressive episodes and follows with a counselor. On examination, she appears lethargic but cooperative. Palpation elicits tenderness over multiple areas, including the trapezius, masseter, and cervical paraspinal muscles. Cervical range of motion is generally preserved in all planes of motion, though she winces slightly with end-range rotation. Temporomandibular joint range of motion was mildly limited in retraction with pain during range of motion and at end range. Neurological screening shows intact strength, reflexes, and sensation in the upper and lower limbs. While performing tandem gait, she demonstrates slight imbalance but quickly recovers, stating she feels “clumsy when tired.” Cranial nerves are grossly intact. |
| negCase_23 | M48.02 — Spinal stenosis, cervical region | A 67-year-old male presents with a history of episodic neck pain over the years and progressive neck discomfort and numbness in both hands that has developed over the past three months. He describes stiffness in the neck and intermittent tingling in all of his fingertips, especially when reading in bed or holding his head in one position for long periods. He denies headache, dizziness, or visual disturbances but notes that his handwriting has recently become more difficult due to a sense of hand clumsiness. His medical history includes hypercholesterolemia, myocardial infarction, and chronic kidney disease stage II. On examination, cervical extension reproduces “pressure-like discomfort” in the lower neck. Triceps reflexes are brisk in the upper extremities, though the remainder are normal, and Hoffman’s sign is present bilaterally. Strength testing shows mild weakness in grip bilaterally. Sensation is diminished to light touch over the fingertips. Tandem gait is unsteady, and he requires correction after a few steps. Finger to nose is poor and rapid alternating movements are uncoordinated. |
| negCase_24 | M48.02 — Spinal stenosis, cervical region | A 70-year-old female presents with a two-month history of neck stiffness and aching discomfort that radiates into the shoulders. She describes the pain as a deep, nagging pressure that worsens when looking upward or holding her head in one position for extended periods. This has impacted her ability to garden and attend her bird watching club. She denies limb weakness, imbalance, or changes in coordination. She also denies headache, dizziness, or visual symptoms. Her past medical history includes well-controlled hypertension, cataracts, and type II diabetes mellitus type. On examination, cervical extension reproduces aching pain in the lower neck and upper shoulders. Neural tension testing is not provocative. Range of motion is moderately limited in extension and rotation. Palpation elicits tenderness along the cervical paraspinal muscles and facet regions. Neurologic screening demonstrates symmetric strength, reflexes, and sensation in the upper extremities. |
| negCase_25 | S06.0 — Concussion | A 24-year-old male presents with neck soreness and headache following a fall during a recreational soccer game three days ago. He reports colliding heads with another player while making a run to the goal. He briefly felt “dazed,” though he did not lose consciousness. Since, he has experienced a persistent dull headache, occasional light sensitivity, and difficulty concentrating on spreadsheets at work. He has been nauseous and dizzy but without vomiting. His past medical history includes surgically corrected right hip dysplasia and otherwise is unremarkable. He does note a couple episodes previously when a similar situation occurred in high school. On examination, he is alert and oriented but appears slightly slowed in his responses to questions and commands. Cervical range of motion is full, with mild discomfort at end-range flexion. Palpation over the paraspinal muscles is mildly tender. Neurologic screening demonstrates intact strength and reflexes in the upper extremities. Cranial nerve examination is grossly normal, though extraocular movements resulted in dizziness, described as “the room is spinning around me.” Tandem gait testing shows mild unsteadiness, though he does not require assistance. |
| negCase_26 | M06.4 — Inflammatory polyarthropathy | A 55-year-old female presents with several weeks of worsening neck pain and stiffness accompanied by aching in both wrists and knees. She describes morning stiffness lasting more than an hour, gradually improving with activity. She denies any recent trauma. She has two aunts with “the rheumatoid.” The neck discomfort is described as a constant dull ache with occasional sharp pain when rotating her head quickly. She also reports fatigue and intermittent low-grade fevers. Her past history includes managed hypothyroidism, managed diabetes type II, and status-post L3-L5 fusion. On examination, there is restricted cervical rotation bilaterally with discomfort at end ranges. Palpation reveals tenderness over the cervical paraspinal region as well as swelling and tenderness of the wrists. Neurologic screening of the upper extremities is unremarkable. Sensation and strength are intact. |
| negCase_27 | M54.12 — Radiculopathy, cervical region | A 39-year-old male presents with right-sided neck pain and pins and needles in his right arm following a fall while mountain biking six weeks ago. He recalls landing hard on his shoulder and immediately noticing a sharp pain radiating from the side of his neck into the right arm. Since then, he has experienced intermittent tingling in the thumb and index finger and describes difficulty holding a coffee mug without discomfort. He did not seek out any medical attention following the fall, stating “I have this all the time, I didn’t need a doctor.” He denies dizziness, vision changes, or gait problems. On examination, cervical extension and right lateral flexion reproduce radiating pain. Spurling’s maneuver on the right increases his neck pain and right arm pins and needles. Strength testing shows mild weakness in elbow flexion compared to the left. Reflexes reveal a reduction at the right biceps, while triceps reflexes are symmetric. Sensation is decreased along the lateral forearm. |
| negCase_28 | S13.4xxA — Sprain of ligaments of cervical spine | A 28-year-old female presents with a four-year history of neck pain that worsened after a minor motor vehicle collision three days ago in which her car was struck from the side. She reports immediate soreness in the lower neck that progressed to be more severe the following morning, describing it as a “dull ache” with “sharp stabs” on quick movements. She denies headache, dizziness, or visual changes. She has a past medical history of Bell’s palsy and recurrent benign positional paroxysmal vertigo responsive to Epley maneuver. On examination, her head carriage is slightly guarded, and cervical rotation is moderately limited bilaterally due to pain, particularly on the left. Palpation elicits tenderness over the mid-cervical spinous processes and adjacent musculature. Neurologic screening shows intact upper extremity strength and reflexes. Sensation is preserved to light touch throughout both arms. |
| negCase_29 | R51.9 — Headache, unspecified | A 50-year-old male presents with a five-day history of intermittent headaches centered over the back of his head and upper neck. He describes the pain as a “steady pressure” that sometimes radiates towards his temples. He notes that on two occasions, he experienced brief episodes of tingling in his left cheek and a sense that his speech “felt heavy,” though both resolved within minutes. He has experienced this a few times over the past four years. He denies persistent weakness, vision loss, or dizziness. His past medical history includes mild hypertension and allergic rhinitis. On examination, he appears mildly uncomfortable but alert. Cervical motion is slightly limited in rotation without clear reproduction of headache symptoms. Neurological screening reveals normal strength and reflexes in the upper extremities. Sensation is intact, though he describes a subtle “numb” feeling along the left cheek when tested with light touch. Otherwise, cranial nerve examination appears normal. |
| negCase_30 | G43.1 — Migraine with aura | A 31-year-old female presents with recurrent, episodic headaches over the past year, the most recent beginning yesterday. She describes a throbbing pain behind her right eye, preceded by shimmering lights in her vision that lasted about 15 minutes. During this episode, she also noticed an unusual brief tingling in her right hand and described her words as “coming out jumbled” for several minutes before the headache fully developed. She denies persistent weakness, facial drooping, or loss of consciousness. Past medical history includes two motor vehicle collisions as a child, generalized anxiety disorder, and post-traumatic stress disorder. On examination, she is fatigued and anxious. She requests the exam room lights be turned down. Palpation of the cervical paraspinal muscles reproduces mild discomfort but does not change the headache. Extraocular movements are intact, though she reports slight blurring when looking far upward. Strength and reflexes in the upper extremities are symmetric, and coordination is preserved. |

ICD-10: International Classification of Disease 10^th^ Edition

References:

1.	Bhavsar D, Gandhi S, Magdum R, Mushtaq I, Giri N. A Case of Horner’s Syndrome Aiding the Diagnosis of Internal Carotid Artery Dissection (ICAD): A Life-Saving Twist of Fate. *Cureus*. Published online April 16, 2024. doi:10.7759/cureus.58367

2.	Buckley KC, Luu A, Grotticelli J, Sahni S. A Workout to Remember: CrossFit-Induced Vertebral Artery Dissection. *Cureus*. Published online June 30, 2023. doi:10.7759/cureus.41211

3.	Futch D, Schneider MJ, Murphy D, Grayev A. Vertebral artery dissection in evolution found during chiropractic examination. *BMJ Case Rep*. 2015;2015:bcr2015212568. doi:10.1136/bcr-2015-212568

4.	Halfpap JP, Cho AA, Rosenthal MD. Cervical Spine Fracture With Vertebral Artery Dissection. *J Orthop Sports Phys Ther*. 2016;46(10):929-929. doi:10.2519/jospt.2016.0416

5.	McCrory P. Vertebral artery dissection causing stroke in sport. *J Clin Neurosci*. 2000;7(4):298-300. doi:10.1054/jocn.1999.0189

6.	Michaud TC. Uneventful upper cervical manipulation in the presence of a damaged vertebral artery. *J Manipulative Physiol Ther*. 2002;25(7):472-483. doi:10.1067/mmt.2002.126468

7.	Mosby JS, Duray SM. Vertebral artery dissection in a patient practicing self-manipulation of the neck. *J Chiropr Med*. 2011;10(4):283-287. doi:10.1016/j.jcm.2011.01.007

8.	Taylor AJ, Kerry R. Neck pain and headache as a result of internal carotid artery dissection: implications for manual therapists. *Man Ther*. 2005;10(1):73-77. doi:10.1016/j.math.2004.06.001

9.	Wityk RJ. Stroke in a Healthy 46-Year-Old Man. *JAMA*. 2001;285(21):2757. doi:10.1001/jama.285.21.2757

10.	Swenson RS. Spontaneous Vertebral Artery Dissection: A Case Report. *J Neuromusculoskelet Syst*. 1993;1(1):10-13.
